# Cytokine Gene Variants Are Associated With Aerobic Fitness‐Related, Hematological, and Metabolic Traits in Healthy Adults

**DOI:** 10.1002/cph4.70234

**Published:** 2026-07-31

**Authors:** Kinga Humińska‐Lisowska, Barkın Bıçakçı, Monika Michałowska‐Sawczyn, Piotr Aschenbrenner, Alison V. September, Patrizia Proia, Agata Leońska‐Duniec

**Affiliations:** ^1^ Faculty of Physical Education Gdansk University of Physical Education and Sport Gdańsk Poland; ^2^ Health Through Physical Activity, Lifestyle, and Sport Research Centre (HPALS), Division of Physiological Sciences, Department of Human Biology University of Cape Town Cape Town South Africa; ^3^ Sport and Exercise Sciences Research Unit, Department of Psychology, Educational Science and Human Movement University of Palermo Palermo Italy

## Abstract

Genetic variation in cytokine genes may influence cytokine‐related signaling, metabolism, and exercise‐related phenotypes, yet large‐scale population data remain scarce. This study examined whether five polymorphisms in interleukin‐6 (IL6; rs1800795; rs1800796; rs1800797), interleukin‐15 (IL15; rs1589241), and tumor necrosis factor‐alpha (TNF‐α; rs1800629) associate with physiological, biochemical, and performance‐related traits in healthy adults. For VO_2_max‐stratified analyses, 501 healthy Polish adults underwent physiological, biochemical, and genomic assessments, classified by maximal oxygen uptake using sex‐and age‐adjusted reference categories. Whole‐cohort analyses were performed for biochemical and hematological traits in up to 962 participants. IL6 rs1800795 CC genotype showed an exploratory higher serum‐iron (*p* = 0.004) and hematocrit (*p* = 0.033). The *IL6* C‐G‐G haplotype (rs1800795‐rs1800796‐rs1800797) was associated with higher serum‐iron (*p* = 0.0012, within‐phenotype FDR‐adjusted *p* = 0.002). *TNF‐α* rs1800629, showed a model‐dependent association with lower aerobic fitness classification in grouped analyses (*p* = 0.006). A‐allele carriers had lower odds of higher‐fitness classification under the dominant model and AG heterozygotes showed the strongest association under overdominant model. Grouped analyses; AG heterozygotes (*p* = 0.037) had higher LDL cholesterol, was not retained in whole‐cohort adjusted‐analyses. *IL15* showed no single‐locus effects but an exploratory multi‐locus genotype combination, *IL15* CC/CT × *TNF‐α* GG, associated with higher odds of aerobic fitness classification (*p* = 0.004). Whole‐cohort analyses: *IL6* rs1800795 was associated with serum‐iron, hematocrit, hemoglobin, and RBC after adjustments. Findings provide preliminary evidence of modest, trait‐specific associations between cytokine‐related genetic variation and hematological, metabolic, and aerobic fitness‐related phenotypes. *IL6* rs1800795 mainly associated with iron‐related and hematological traits; *TNF‐α* rs1800629 findings were model‐dependent and inconsistent. *IL15* CC/CT × *TNF‐α* GG combination is exploratory, requiring independent replication.

## Graphical abstract

Analysis of ~1000 adults shows cytokine gene variants are more strongly associated with hematological and metabolic traits than fitness classification. IL6 and TNF‐α variants link to iron status and aerobic fitness, with exploratory multi‐locus effects involving IL15.

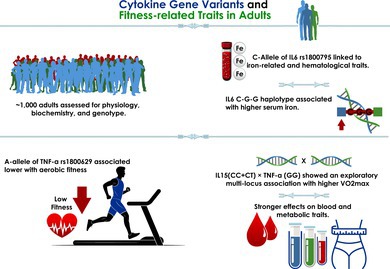

## 1 Introduction

The positive impact of physical activity on reduced mortality, decreased risk of developing non‐communicable diseases, and improved mental health has been well recognized for decades (Humińska‐Lisowska 2024; Warburton and Bredin 2017). Despite this, the mechanisms that translate repeated bouts of exercise into durable adaptations are not fully understood. A central working model posits an inflammatory‐regenerative axis in which acute, contraction‐induced signals trigger systemic communication that drives remodeling and performance improvements. However, searching for a muscle contraction‐induced humoral factor, an “exercise factor” that could cause some of the exercise‐induced changes in major metabolic organs represents a novel challenge. Recent research has revealed that skeletal muscle in response to external stimuli can secrete a group of biochemical molecules usually known as myokines (Pedersen et al. 2003, 2007). In general, myokines are cytokines and other peptides that are produced, expressed, and released by muscle fibers and regulate whole‐body metabolism in an autocrine, paracrine, or endocrine manner (Pedersen et al. 2007; Chen et al. 2021). The conceptual basis of considering these products as key mediators within the complex organ communication network is essential for developing a novel understanding of the molecular basis of physical activity (Eckel 2019). This framework is in line with emerging evidence that biomarkers of tissue stress and inflammation can be used to monitor training load and recovery.

Although it had long been suggested, it was ~25 years ago that the first proposed muscle contraction‐induced factor mediating exercise effects in other organs, interleukin‐6 (IL6), was described by Pedersen et al. (2001). Ongoing research demonstrates that skeletal muscle tissue may produce inflammatory biomarkers belonging to different families such as interleukin‐15 (IL15) or tumor necrosis factor‐alpha (TNF‐α) (Pedersen et al. 2007; Nielsen et al. 2007; Petersen and Pedersen 2005). These interleukins potentially have anabolic effects in human skeletal muscle, can be associated with hypertrophy muscle growth, play an important role in regulating energy metabolism, and consequently training adaptation (Pedersen et al. 2007; Folgueira et al. 2024). Convincing evidence exists that the expression of *IL6* and *IL15* is regulated by a bout of exercise (Nielsen et al. 2007; Febbraio and Pedersen 2002). In addition, *IL6* expression during muscle contraction may inhibit the production of the proinflammatory cytokine TNF‐α, which is significantly involved, among others, in glucose metabolism (Petersen and Pedersen 2005). Our group has also demonstrated that systemic biomarkers (cfDNA, adipokines) respond to exercise intensity and body composition, supporting the relevance of inflammation‐related signals in training (Humińska‐Lisowska et al. 2021, 2022). Taken together, IL6, IL15, and TNF‐α form a biologically plausible and tractable triad through which the inflammatory‐regenerative axis influences phenotype and performance. However, interindividual variability in myokine production and release has been observed due to functional single nucleotide polymorphisms (SNPs) in the genes encoding these molecules. These genetic variants may affect gene transcription and cytokine synthesis, altering the intensity of the inflammatory response, individual susceptibility to certain diseases, as well as the effectiveness of exercise training programs (De Maat et al. 2004). As genetics are thought to influence between 44% and 64% of variability in VO_2max_ (Bıçakçı et al. 2024). Therefore, genetic modulation of myokine signaling is a plausible cause of the differences between people in how they adapt to training stimuli.

Although almost 50 SNPs have been described for *IL6* gene (7p15.3) so far (Blumenfeld et al. 2014), the SNPs located in the promoter region seem to be particularly important because they may quantitatively change the gene's expression (Akhter et al. 2019). Thus, three promoter *IL6* polymorphisms, namely, rs1800795 (−174G>C), rs1800796 (−572G>C), and rs1800797 (−597A>G), associated with *IL6* transcription activity, were selected for this study. In addition, to get a better picture of the complex interactions among various genetic variants, SNPs rs1589241 (T>A) in the regulatory element (first intron, 3′UTR) of *IL15* (4q31.21) and rs1800629 (−308G>A) in the promoter region of *TNF‐α* (6p21.33) were added to the analysis. It is noteworthy that only simultaneous analysis of numerous polymorphic sites can provide additional unique information about the associations between genetic background and phenotypic traits, as well as insight into the dependency among genetic markers (Liu et al. 2008). Therefore, we prioritized a parsimonious and biologically grounded candidate panel spanning myokine production (IL6 and IL15) and proinflammatory tone (TNF‐α).

Given that genetic variations may affect cytokine‐related signaling, we hypothesized that polymorphic sites in the *IL6*, *IL15*, and *TNF‐α* genes could be associated with body composition, selected hematological and biochemical parameters, and variables of aerobic and anaerobic performance. The present study therefore aimed to investigate both the individual and combined roles of five SNPs—three in the *IL6* promoter region (rs1800795, rs1800796, and rs1800797), one in *IL15* (rs1589241), and one in *TNF*‐α (rs1800629)—in Polish adults aged 20–57 with varying levels of aerobic fitness. Specifically, we examined whether these SNPs are associated with differences in body composition, selected blood parameters, and both aerobic and anaerobic exercise capacity. Analyses were performed both in VO_2_max‐stratified fitness groups and, for selected traits, across the whole cohort to evaluate continuous genotype–phenotype associations. Because these proinflammatory and myokine‐related genes may underlie individual variability in training response and exercise adaptation, we further explored potential interactions among these variants (e.g., *IL6* haplotypes combined with *IL15* and *TNF‐α* genotypes) to determine whether specific genetic profiles coincide with the observed variation in muscle‐related traits and metabolic indices. This cross‐sectional study is part of a broader research program examining the inflammatory‐regenerative axis of exercise adaptation using circulating biomarkers, microbiome features, and genetics. The present analysis focuses specifically on the genetic component of this framework.

## 2 Materials and Methods

### 2.1 Study Design and Participants

This cross‐sectional study was part of the internal financial program at the University of Physical Education and Sport in Gdańsk and co‐financed from the state budget under the program of the Minister of Education and Science, “Science for Society II”. All measurements and biological sampling were performed at a single study visit (one time point) with no longitudinal follow‐up. Of the 1000 adults of self‐reported Polish ancestry who were recruited, 964 were enrolled after eligibility screening and exclusions. Primary analyses included only individuals with complete maximal oxygen uptake (VO_2_max), cardiovascular/hematological, and genotyping data (*n* = 860). For selected whole‐cohort analyses of biochemical and hematological phenotypes, the analytical sample varied by outcome depending on data completeness and included up to 962 participants with genotype and relevant phenotype data.

The inclusion criteria were as follows: age between 20 and 57 years, good general health, absence of a diagnosed cardiovascular, metabolic, autoimmune, or neurological disease, no current use of anti‐inflammatory, hormonal, or performance‐enhancing medications, written informed consent, completion of standardized physiological testing including VO_2_max, and provision of venous blood, buccal swab, and stool samples. The exclusion criteria were missing VO_2_max, use of drugs that affect inflammatory, endocrine, or cardiometabolic function, acute injury or infection within the previous 3 months, and genetic data that failed quality control.

Participants attended a cooperating hospital in a fasted state for screening (fasting blood and urine tests, chest X‐ray). Eligible individuals received home sampling kits for saliva, stool, oral swabs, and urine, and were scheduled for laboratory assessments. Each participant was assigned an anonymized study code. The study complied with the Declaration of Helsinki and was approved by the Bioethics Committee at the District Medical Chamber in Gdańsk (KB‐16/20). Written informed consent was obtained for biospecimen collection, genetic analyses, and use of anonymized data.

#### 2.1.1 Fitness Classification

Finally, for the grouped VO_2_max‐stratified analyses, 501 participants with complete physiological (including VO_2_max), biochemical, and genotyping data were included. To objectively assess fitness status, participants were stratified based upon their VO_2_max into predefined sex‐ and age‐adjusted categories adapted from Shvartz and Reibold. Categories were very poor, poor, satisfactory, medium, good, very good, and outstanding. For group‐based comparisons, these categories were collapsed into two VO_2_max‐defined fitness strata: High Fitness (HF; very good and outstanding) and Low Fitness (LF; very poor, poor, and satisfactory). Participants classified as medium or good were excluded from HF–LF comparisons and were not used as a control group. This extreme‐phenotype approach was used to maximize separation between the two fitness strata in the grouped analysis. It was not intended to represent the full continuum of aerobic fitness, which is acknowledged as a limitation of the HF–LF comparison.

### 2.2 Anthropometric, Body Composition, and Performance Testing

Anthropometry and body composition were assessed under standardized conditions. Stature was measured to 0.1 cm with a wall stadiometer and body mass was measured to 0.1 kg with a calibrated digital scale. Body composition, including fat mass, fat‐free mass, and total body water, was obtained by multi‐frequency bioelectrical impedance analysis using an InBody 720 (Biospace, Seoul, South Korea). Waist and hip circumferences were measured with a non‐elastic tape according to the World Health Organization protocol. Body mass index (BMI) was calculated as weight divided by height squared (kg/m^2^). Testing occurred in a fasted state or at least 3 h after a light meal, consistent with bioimpedance guidelines.

Aerobic performance was evaluated using an incremental cycling test to volitional exhaustion with breath‐by‐breath gas analysis (Jaeger Oxycon Pro, CareFusion, Höchberg, Germany) on an electronically braked ergometer (Ergoline Ergoselect viasprint 150p, Bitz, Germany). After a five‐minute warm‐up at 60 rpm with a load of 1.0 W·kg^−1^, the protocol consisted of two‐minute stages, beginning at 1.5 W·kg^−1^ with 0.5 W·kg^−1^ increments per stage, until exhaustion. Oxygen uptake (VO_2_), carbon dioxide output (VCO_2_), respiratory exchange ratio (RER), and minute ventilation (VE) were recorded continuously. Heart rate (HR) was monitored using a Polar H9 chest strap.

Anaerobic performance was evaluated using a 30‐s Wingate test on a friction‐loaded cycle ergometer (Monark 894E, Sweden). The braking force was set at 0.075 kg per kilogram of body mass. Peak and mean power were expressed relative to body mass (W·kg^−1^).

### 2.3 Biochemical and Hematological Analyses

Trained personnel at the cooperating hospital (7th Navy Hospital in Gdańsk) drew fasting venous blood (early morning between 7:15 and 10:00), into EDTA and serum tubes and processed it according to standard operating procedures. Analyses were performed at a certified clinical laboratory (Diagnostyka Sp. z o.o.) using automated analyzers.

A complete blood count includes the following: white blood cell count (WBC), red blood cell count (RBC), hemoglobin (HGB), hematocrit (HCT), mean corpuscular volume (MCV), mean corpuscular hemoglobin (MCH), mean corpuscular hemoglobin concentration (MCHC), and platelets (PLT).

Serum biochemistry: glucose, creatinine, iron, cortisol, total cholesterol (TC), high‐density lipoprotein (HDL), low‐density lipoprotein (LDL), and triglycerides (TG). Circulating inflammatory cytokines (e.g., interleukins and TNF‐α protein concentrations) were not measured in this study.

### 2.4 DNA Isolation, Genotyping, and Statistical Analysis

Genomic deoxyribonucleic acid (DNA) was extracted from buccal epithelial cells collected with sterile FLOQSwabs (Copan, Brescia, Italy) using the High Pure PCR Template Preparation Kit (Roche, Mannheim, Germany), following the manufacturer's instructions. The genotyping targeted the following variants: *interleukin*‐6 (*IL6*) promoter variants rs1800795 (−174G>C, assay ID C___1839697_20), rs1800796 (−572G>C, C__11326893_10), and rs1800797 (−597A>G, C___1839695_20); *interleukin‐15* (*IL15*) variant rs1589241 (C___8865652_10); and *tumor necrosis factor‐alpha* (*TNF‐α*) variant rs1800629 (−308G>A, C___7514879_10). Allelic discrimination was performed using TaqMan SNP Genotyping Assays (Applied Biosystems, CA, USA) on a real‐time polymerase chain reaction (PCR) platform (Bio‐Rad CFX96, CA, USA).

Each 5‐μL reaction contained the following: 2.5 μL of TaqPath ProAmp Master Mix; 0.25 μL of a 10× SNP assay mix containing VIC/FAM‐labeled probes; 1.0 μL of nuclease‐free water; and 1.0 μL of genomic DNA. The thermocycling protocol included a pre‐read at 60°C for 30 s, an initial denaturation at 95°C for 5 min, and 40 cycles of denaturation at 95°C for 5 s and annealing/extension at 60°C for 30 s. A post‐read at 60°C for 30 s was also included. Fluorescence clusters were visualized and genotype calls were reviewed in CFX Maestro software (Bio‐Rad, version 2.3, CA, USA). Quality control included visual inspection of fluorescence clusters and review of genotype calls in CFX Maestro software. Samples and markers with call rates below 95% were excluded. Each 96‐well plate included at least two duplicate samples, and duplicate samples showed 100% concordance. Hardy–Weinberg equilibrium was assessed using an exact test in the pooled sample and within fitness categories. Samples or genotype calls not meeting the applicable quality‐control criteria were excluded before statistical analysis.

### 2.5 Statistical Analysis

Group differences by genotype were examined using Kruskal–Wallis rank sum tests for continuous traits. Initial screening analyses across phenotypes were considered exploratory. Linear models and analysis of covariance (ANCOVA) were adjusted for age, sex, and BMI, and VO_2_max‐based fitness categories were derived from sex‐ and age‐adjusted normative standards to minimize age‐related confounding. Single‐locus associations were evaluated under predefined inheritance models (codominant, dominant, recessive, overdominant, and log‐additive). For grouped VO_2_max‐based analyses, logistic regression was used to estimate odds ratios and 95% confidence intervals for High Fitness versus Low Fitness status. For selected whole‐cohort continuous phenotypes, inheritance‐model analyses were additionally performed with adjustment for age, sex, and BMI. Hardy–Weinberg equilibrium and association analyses were performed in R (Koné 2024) using the SNPassoc package (version 2.1‐2, R Foundation for Statistical Computing, Vienna, Austria). Results are presented with effect estimates and 95% CIs where applicable, and two‐sided *p* values. Statistical reporting adhered to the CHAMP checklist for transparency and rigor (e.g., model specification, variable coding, assumption checks, and handling of small cell counts), with the CHAMP statement (Mansournia et al. 2021).

To address multiple‐testing inflation, analyses were organized in a hierarchical framework. Targeted single‐locus follow‐up analyses included associations of IL6 rs1800795 with serum iron and hematological traits, and of TNF‐α rs1800629 with VO_2_max fitness group. These findings were interpreted according to the relevant nominal, covariate‐adjusted, or Benjamini–Hochberg false discovery rate‐adjusted *p* values, as specified in the corresponding tables. All remaining single‐locus screening analyses, the exploratory IL6 haplotype screen across 43 traits, targeted gene–gene interaction analyses, and the logic‐regression multi‐locus analysis were considered exploratory and hypothesis‐generating. Unless otherwise stated, unadjusted results are reported as nominal *p* values, adjusted‐model *p* values refer to models adjusted for age, sex, and BMI, and Q values refer to Benjamini–Hochberg FDR‐adjusted *p* values.

#### 2.5.1 IL‐6 Haplotype‐Based Analysis

Haplotype‐based *IL6* association analyses were conducted in two steps. A targeted subset of phenotypes showing single‐locus evidence of association (serum iron, HCT, HGB, RBC count) were tested using a hierarchical approach: an omnibus *IL6* haplotype test for each phenotype (using haplo.score), and for phenotypes with significant global evidence, haplotype‐specific regression models (using haplo.glm function) to obtain effect estimates (adjusted for age, sex). These targeted follow‐up analyses were hypothesis‐oriented but should not be interpreted as confirmatory because phenotype selection was informed by the initial single‐locus screening. Multiple testing across primary phenotypes was controlled using the Benjamini–Hochberg false discovery rate (FDR) applied to omnibus *p* values, within any significant phenotype, haplotype‐specific *p*‐values were FDR‐adjusted. To screen remaining phenotypes, we repeated the omnibus *IL6* haplotype test. These results were error controlled using BH‐FDR across all omnibus tests in this step. Follow‐up haplotype‐specific effects were only summarized for exploratory phenotypes passing the FDR threshold. Haplo.score and haplo.glm functions are available in the haplo.stats R package (version 1.9.7, R Foundation for Statistical Computing, Vienna, Austria).

#### 2.5.2 Targeted Gene–Gene Interaction

Targeted gene–gene interaction analyses were performed for selected phenotypes showing single‐locus signals or biological relevance to the study aims, including serum iron, HCT, LDL cholesterol, and mean VO_2_max. These analyses were exploratory. For each selected phenotype we added a block of *IL6* × *IL15* (and separately *IL6* × *TNF‐α*) interaction terms for the most frequent (> 1% for continuous and > 10% for VO_2_max) non‐reference *IL6* haplotypes (haplo.glm) and compared models with and without these terms using block likelihood‐ratio tests. Multiple testing was controlled using the Benjamini–Hochberg FDR (Q value threshold 0.100), while the nominal significance was set at *p* < 0.05.

#### 2.5.3 Data‐Driven Model Free Approach

To analyze higher‐order and non‐additive interactions among gene variants, we applied a logic regression framework integrating SNPs from the *IL6*, *IL15*, and *TNF* loci. Logic regression is a model‐free, data‐driven approach that identifies combinations of binary predictors that best explain categorical outcome. The analysis was conducted using the LogicFS R package (version 2.22.0 Bioconductor Project, Seattle, USA). Each SNP was represented by two binary dummy variables to capture all three genotypic states: SNP_1 = 0, SNP_2 = 0 for homozygous reference, SNP_1 = 1, SNP_2 = 0 for heterozygous; and SNP_1 = 1, SNP_2 = 1 for homozygous variant. To prevent overfitting and for evaluation purposes, we performed 100 bootstrap runs on real data and 100 runs on phenotype‐resampled (VO_2_max status shuffled) data to obtain null distributions. For each run, importance scores were computed to quantify the contribution of individual genotype combinations to model performance. Combinations that occurred in more than 10% of real‐data iterations were retained for further evaluation. Bootstrap‐based 95% confidence intervals for importance scores were estimated to assess stability and significance relative to the resampled data.

### 2.6 Equity, Diversity, and Inclusion Statement

This study was designed and conducted in alignment with the principles of equity, diversity, and inclusion (EDI). We recruited adults from across Poland (multiple regions and urban/rural settings) and aimed for balance by sex and age within the eligibility constraints of a healthy, physically active cohort. Study visits were scheduled to accommodate work/study hours, with clear plain‐language materials and standardized procedures to support inclusive participation. Analyses included sex‐ and age‐adjusted models and we report sex distribution and key demographics; we do not generalize beyond community‐dwelling European adults and acknowledge this as a limitation. The multidisciplinary author team includes investigators at various academic levels (junior and senior researchers) and reflects collaboration across physiology, sports medicine, genetics, and biostatistics.

## 3 Results

Participants were assigned to seven predefined VO_2_max categories according to sex and age (adapted from Shvartz and Reibold (1990), Table S1). For grouped analyses, categories were dichotomized into High Fitness (HF: very good, outstanding, *N* = 311) and Low Fitness (LF: very poor, poor, satisfactory, *N* = 190). Participants classified as medium or good (*N* = 359) were excluded from the HF/LF comparison. Baseline biochemical, hematological, anthropometric, and performance differences between the HF and LF groups are presented in Table 1.

**TABLE 1:** Baseline biochemical, hematological, anthropometric, and performance characteristics by aerobic fitness group (Low fitness, LF vs. High fitness, HF).

Characteristic		LF *N* = 190^a^	HF *N* = 311^a^	*p* ^b^
Glucose (mg·dL^−1^)		98 (11)	94 (9)	**< 0.001**
Creatinine (mg·dL^−1^)		0.91 (0.16) (*N* = 189)	0.89 (0.14) (*N* = 310)	0.263
Iron (μg·dL^−1^)		111 (47) (*N* = 189)	119 (46) (*N* = 310)	0.057
WBC (10^9^·L^−1^)		6.33 (1.68)	5.23 (1.34)	**< 0.001**
RBC (10^12^·L^−1^)		5.00 (0.42)	4.84 (0.42)	**< 0.001**
HGB (g·dL^−1^)		14.93 (1.31)	14.59 (1.17)	**0.001**
HCT (%)		44.04 (3.28)	43.37 (3.07)	**0.021**
MCV (fL)		88.2 (3.8)	89.8 (3.8)	**< 0.001**
MCH (pg)		29.88 (1.52)	30.19 (1.42)	**0.009**
MCHC (g·dL^−1^)		33.89 (0.96)	33.64 (0.86)	**< 0.001**
PLT (10^9^·L^−1^)		239 (50) (*N* = 189)	231 (48)	**0.044**
TC (mg·dL^−1^)		195 (37) (*N* = 188)	187 (35)	**0.028**
HDL (mg·dL^−1^)		56 (15) (*N* = 189)	74 (17)	**< 0.001**
LDL (mg·dL^−1^)		125 (36) (*N* = 189)	112 (32)	**< 0.001**
TG (mg·dL^−1^)		131 (80) (*N* = 189)	81 (36) (*N* = 310)	**< 0.001**
Cortisol (μg·dL^−1^)		15.2 (5.4) (*N* = 189)	16.3 (5.3)	**0.029**
Body mass (kg)		86 (17)	72 (11)	**< 0.001**
Waist circumference (cm)		92 (13)	79 (8)	**< 0.001**
Hip circumference (cm)		103 (8)	96 (5)	**< 0.001**
BMI (kg·m^−2^)		26.9 (4.5)	23.1 (2.3)	**< 0.001**
HR (beats·min^−1^)		75 (14) (*N* = 189)	67 (11)	**< 0.001**
Sex				0.031
Females		50/190 (26%)	112/311 (36%)	
Males		140/190 (74%)	199/311 (64%)	
Age (years)		35 (8)	36 (8)	**0.011**
Stage of test termination		3.81 (0.98)	6.74 (1.33)	**< 0.001**
Time of test termination (min)		11.2 (1.9)	17.0 (2.6)	**< 0.001**
HRmax (beats·min^−1^)		177 (15)	181 (11)	**0.001**
VO_2_max (mL/(min/kg))		33 (5)	53 (7)	**< 0.001**
VO_2_max (L·min^−1^)		2.79 (0.65)	3.81 (0.85)	**< 0.001**
VEmax (L·min^−1^)		107 (28)	143 (35)	**< 0.001**
RER_max		1.22 (0.08)	1.18 (0.06)	**< 0.001**
Workload at termination (W/kg)		2.90 (0.49)	4.38 (0.66)	**< 0.001**
End of test		14.2 (1.9)	20.0 (2.6)	**< 0.001**
Mean		0.78 (0.08)	0.78 (0.09)	0.571
Minimum		0.49 (0.07)	0.48 (0.06)	**0.036**
Maximum		1.67 (0.99)	1.59 (0.62)	0.539
Fatigue index		0.02 (0.13) (*N* = 182)	0.02 (0.11) (*N* = 299)	0.849
Average power (W)		555 (150)	552 (133)	0.586
Relative average power (per body mass) (W·kg^−1^)		6.47 (1.15)	7.64 (1.06)	**< 0.001**
Maximum power (W)		735 (201)	680 (173)	**0.001**
Relative maximum power (per body mass) (W·kg^−1^)		8.53 (1.47)	9.39 (1.40)	**< 0.001**
Time to reach P_max_ (s)		6.42 (3.26)	6.71 (2.18)	**< 0.001**
Maximum rotational speed (rev·s^−1^)		1.95 (0.35)	2.13 (0.32)	**< 0.001**
Strength–speed index		97 (42)	80 (31)	**< 0.001**

*Note:* Bold values indicate statistically significant results at *p* < 0.05.

Abbreviations: Average power (W) and relative average power (W·kg^−1^); End of test, rating of perceived exertion (Borg 6–20); Fatigue index, Wingate‐derived fatigue metric; HCT, hematocrit (%); HDL, high‐density lipoprotein cholesterol (mg·dL^−1^); HF, High Fitness; HGB, hemoglobin (g·dL^−1^); HR, heart rate (beats·min^−1^); HRmax, maximal heart rate (beats·min^−1^); LDL, low‐density lipoprotein cholesterol (mg·dL^−1^); LF, Low Fitness; Maximum power (W) and relative maximum power (W·kg^−1^); Maximum rotational speed, (rev·s^−1^); MCH, mean corpuscular hemoglobin (pg); MCHC, mean corpuscular hemoglobin concentration (g·dL^−1^); MCV, mean corpuscular volume (fL); PLT, platelets (10^9^·L^−1^); RBC, red blood cell count (10^12^·L^−1^); RER_max, maximal respiratory exchange ratio; Stage/Time of test termination, stage number and elapsed time (min) at cessation of the incremental test; TC, total cholesterol (mg·dL^−1^); TG, triglycerides (mg·dL^−1^); Time to reach P_max_, time to peak power (s); VEmax, maximal minute ventilation (L·min^−1^); VO_2_max, maximal oxygen uptake reported as mL·kg^−1^·min^−1^ (mass‐normalized) and L·min^−1^ (absolute); WBC, white blood cell count (10^9^·L^−1^); Workload at termination, final incremental load (W·kg^−1^).

^a^ *n*/*N* (%) or mean (SD).

^b^ Pearson's Chi‐squared test; Kruskal–Wallis rank sum test.

All SNPs conformed to Hardy–Weinberg equilibrium (HWE) within LF and HF groups except *TNF‐α* rs1800629 in the HF group (*p* = 0.034). In the pooled sample, *IL15* rs1589241 (*p* = 0.022) and *IL6* rs1800797 (*p* = 0.00018) deviated from HWE. These departures from HWE are considered in the Limitations section, and findings involving these variants are interpreted cautiously.

### 3.1 Single Locus‐Analysis

Across the assessed domains, including body composition (bioimpedance: skeletal muscle mass, fat mass, body‐fat percentage), blood parameters (CBC, lipid profile, glucose, creatinine, iron, cortisol), and performance tests (VO_2_max on cycle ergometer; Wingate peak and mean power), we identified several nominal genotype–phenotype associations in the initial exploratory screening analyses. Specifically, *IL6* rs1800795 was associated with serum iron (*p* = 0.004) and hematocrit (*p* = 0.033), whereas *TNF‐α* rs1800629 was associated with fitness group (LF vs. HF; *p* = 0.006) and LDL cholesterol (*p* = 0.037) (Tables S2–S6). *IL15* rs1589241 did not show any nominal associations within the final set of outcomes. The association between IL6 rs1800795 and serum iron was subsequently evaluated under alternative inheritance models (Table 2).

**TABLE 2:** Association of *IL6* rs1800795 with serum iron (μg·dL^−1^) in the whole cohort.

Model	*n*	Me	Se	Dif	Lower	Upper	*p* ^a^
Codominant							
G/G	292	108.32	2.77	0.00			**0.030**/0.067
C/G	473	117.41	2.23	9.10	2.18	16.01	
C/C	195	111.57	3.22	3.26	−5.34	11.86	
Dominant							
G/G	292	108.32	2.77	0.00			**0.027/0.040**
C/G‐C/C	668	115.71	1.84	7.39	0.86	13.92	
Recessive							
G/G‐C/G	765	113.94	1.74	0.00			0.536/0.741
C/C	195	111.57	3.22	−2.36	−9.85	5.12	
Overdominant							
G/G‐C/C	487	109.62	2.10	0.00			**0.011/0.031**
C/G	473	117.41	2.23	7.79	1.79	13.79	
Log‐additive							
0,1,2				2.38	−1.89	6.64	0.275/0.250

*Note:* COD (codominant: G/G vs. C/G vs. C/C; reference = G/G), DOM (dominant: C/G–C/C vs. G/G; reference = G/G), REC (recessive: C/C vs. G/G–C/G; reference = G/G–C/G), OVER (overdominant: C/G vs. G/G–C/C; reference = G/G–C/C), Log‐additive (0,1,2) = per‐allele effect coded by C‐allele dosage. 0 corresponds to a reference genotype, lower/upper—confidence intervals for difference in mean. Bold values indicate statistically significant results at *p* < 0.05.

Abbreviations: diff = mean difference, me = estimated marginal mean, *n* = sample size, se = standard error.

^a^ *p* values based on rank‐transformed values, after slash—*p* values adjusted for age, BMI and sex.

In addition to the grouped HF/LF analyses, we further examined selected whole‐cohort associations for *IL6* rs1800795 using inheritance models adjusted for age, BMI, and sex. In these analyses, rs1800795 was associated with serum iron under the dominant (adjusted *p* = 0.040) and overdominant (adjusted *p* = 0.031) models, with hematocrit under the codominant (adjusted *p* = 0.029), dominant (adjusted *p* = 0.008), and log‐additive (adjusted *p* = 0.015) models, with hemoglobin under the dominant model (adjusted *p* = 0.020), and with RBC count under codominant (adjusted *p* = 0.0013), dominant (adjusted *p* = 0.0003), and overdominant (adjusted *p* = 0.025) models (Tables 3, 4, 5). These outcome‐specific whole‐cohort analyses (*N* = 962) indicate that *IL6*‐related variation was more consistently associated with hematological traits than with dichotomized fitness status itself.

**TABLE 3:** Association of *IL6* rs1800795 with hematocrit (HCT, %) in the whole cohort.

Model	*n*	Me	Se	Dif	Lower	Upper	*p* ^a^
Codominant							
G/G	292	43.17	0.18	0.00			**0.036/0.029**
C/G	473	43.71	0.14	0.54	0.10	0.98	
C/C	196	43.74	0.23	0.58	0.03	1.12	
Dominant							
G/G	292	43.17	0.18	0.00			**0.010/0.008**
C/G‐C/C	669	43.72	0.12	0.55	0.13	0.97	
Recessive							
G/G‐C/G	765	43.50	0.11	0.00			0.321/0.205
C/C	196	43.74	0.23	0.24	−0.24	0.72	
Overdominant							
G/G‐C/C	488	43.40	0.14	0.00			0.117/0.160
C/G	473	43.71	0.14	0.31	−0.08	0.69	
Log‐additive							
0,1,2				0.31	0.04	0.58	**0.025/0.015**

*Note:* COD (codominant: G/G vs. C/G vs. C/C; reference = G/G), DOM (dominant: C/G–C/C vs. G/G; reference = G/G), REC (recessive: C/C vs. G/G–C/G; reference = G/G–C/G), OVER (overdominant: C/G vs. G/G–C/C; reference = G/G–C/C), log‐additive (0,1,2) = per‐allele effect coded by C‐allele dosage. 0 corresponds to a reference genotype, lower/upper—confidence intervals for difference in mean. Bold values indicate statistically significant results at *p* < 0.05.

Abbreviations: diff = mean difference, me = estimated marginal mean, *n* = sample size, se = standard error.

^a^ *p* values based on rank‐transformed values, after slash—*p* values adjusted for age, BMI and sex.

**TABLE 4:** Association of *IL6* rs1800795 with hemoglobin (HGB) in the whole cohort.

Model	*n*	Me	Se	Dif	Lower	Upper	*p* ^a^
Codominant							
G/G	292	14.58	0.07	0.00			0.079/0.068
C/G	473	14.77	0.05	0.19	0.01	0.37	
C/C	196	14.77	0.09	0.20	−0.02	0.42	
Dominant							
G/G	292	14.58	0.07	0.00			**0.024/0.020**
C/G‐C/C	669	14.77	0.05	0.19	0.03	0.36	
Recessive							
G/G‐C/G	765	14.69	0.04	0.00			0.423/0.413
C/C	196	14.77	0.09	0.08	−0.11	0.27	
Overdominant							
G/G‐C/C	488	14.65	0.06	0.00			0.155/0.141
C/G	473	14.77	0.05	0.11	−0.04	0.27	
Log‐additive							
0,1,2				0.11	−0.00	0.22	0.054/**0.048**

*Note:* COD (codominant: G/G vs. C/G vs. C/C; reference = G/G), DOM (dominant: C/G–C/C vs. G/G; reference = G/G), REC (recessive: C/C vs. G/G–C/G; reference = G/G–C/G), OVER (overdominant: C/G vs. G/G–C/C; reference = G/G–C/C), log‐additive (0,1,2) = per‐allele effect coded by C‐allele dosage. 0 corresponds to a reference genotype, lower/upper—confidence intervals for difference in mean. Bold values indicate statistically significant results at *p* < 0.05.

Abbreviations: diff = mean difference, me = estimated marginal mean, *n* = sample size, se = standard error.

^a^ *p* values based on rank‐transformed values, after slash—*p* values adjusted for age, BMI and sex.

**TABLE 5:** Association of *IL6* rs1800795 with red blood cell count (RBC) in the whole cohort.

Model	*n*	Me	Se	Dif	Lower	Upper	*p* ^a^
Codominant							
G/G	292	4.86	0.02	0.00			**0.017/0.0013**
C/G	473	4.94	0.02	0.08	0.02	0.14	
C/C	196	4.94	0.03	0.08	0.01	0.15	
Dominant							
G/G	292	4.86	0.02	0.00			**0.004/0.0003**
C/G‐C/C	669	4.94	0.02	0.08	0.03	0.14	
Recessive							
G/G‐C/G	765	4.91	0.01	0.00			0.359/0.174
C/C	196	4.94	0.03	0.03	−0.03	0.09	
Overdominant							
G/G‐C/C	488	4.89	0.02	0.00			0.059/**0.025**
C/G	473	4.94	0.02	0.05	−0.00	0.10	
Log‐additive							
0,1,2				0.04	0.01	0.08	**0.017**

*Note:* COD (codominant: G/G vs. C/G vs. C/C; reference = G/G), DOM (dominant: C/G–C/C vs. G/G; reference = G/G), REC (recessive: C/C vs. G/G–C/G; reference = G/G–C/G), OVER (overdominant: C/G vs. G/G–C/C; reference = G/G–C/C), log‐additive (0,1,2) = per‐allele effect coded by C‐allele dosage. 0 corresponds to a reference genotype, lower/upper—confidence intervals for difference in mean. Bold values indicate statistically significant results at *p* < 0.05.

Abbreviations: diff = mean difference, me = estimated marginal mean, *n* = sample size, se = standard error.

^a^ *p* values based on rank‐transformed values, after slash—*p* values adjusted for age, BMI and sex.

In grouped‐model analyses, *IL6* rs1800795 remained associated with serum iron across most inheritance models, except the recessive model (codominant: *p* = 0.006, FDR‐adjusted [Q] = 0.015, dominant: *p* = 0.002, Q = 0.010, overdominant: *p* = 0.009, Q = 0.015, log‐additive: *p* = 0.029, Q = 0.036, recessive: *p* = 0.783, Q = 0.783, Table 6), whereas associations with hematocrit were weaker and retained significant only under the dominant model: *p* = 0.020, Q = 0.080, and log‐additive model: *p* = 0.032, Q = 0.080 (Table 7).

**TABLE 6:** Association of *IL6* rs1800795 with serum iron (μg·dL^−1^) in the VO_2_max defined high fitness and low fitness groups.

	Model	*n*	Me (SE)	Diff (95% CI)	*p* ^a^
COD	G/G	150	105.3 (3.4)	0.0	**0.004/0.006**
	C/G	250	122.3 (3.1)	16.9 (7.6; 26.3)	
	C/C	99	116.5 (4.6)	11.2 (−0.6; 22.9)	
DOM	G/G	150	105.3 (3.4)	0.0	**0.001/0.002**
	C/G‐C/C	349	120.6 (2.6)	15.3 (6.5; 24.1)	
REC	G/G‐C/G	400	115.9 (2.4)	0.0	0.762/0.783
	C/C	99	116.5 (4.6)	0.58 (−9.69; 10.85)	
OVER	G/G‐C/C	249	109.8 (2.8)	0.0	**0.007/0.009**
	C/G	250	122.3 (3.1)	12.5 (4.4; 20.6)	
Log‐additive 0,1,2				6.77 (0.94; 12.60)	**0.023/0.029**

*Note:* COD = codominant model (G/G vs. C/G vs. C/C; reference = G/G); DOM = dominant model (C/G–C/C vs. G/G; reference = G/G); REC = recessive model (C/C vs. G/G–C/G; reference = G/G–C/G); OVER = overdominant model (C/G vs. G/G–C/C; reference = G/G–C/C); log‐additive (0,1,2) = per‐allele effect coded by C‐allele dosage. Bold values indicate statistically significant results at *p* < 0.05.

Abbreviations: dif = mean difference, lower and upper = lower and upper bounds of the 95% confidence interval, me = estimated marginal mean, *n* = sample size, se = standard error.

^a^ Values before the slash represent *p* values based on rank‐transformed data; values after the slash represent *p* values adjusted for age, BMI, and sex.

**TABLE 7:** Association of *IL6* rs1800795 with hematocrit (HCT, %) in the VO_2_max‐defined high fitness and low fitness groups.

Model		*n*	Me (SE)	Dif (95% CI)	*p* ^a^
COD	G/G	151	43.04 (0.26)	0.0	**0.033**/0.063
	C/G	250	43.96 (0.19)	0.92 (0.29; 1.56)	
	C/C	100	43.69 (0.35)	0.65 (−0.14; 1.44)	
DOM	G/G	151	43.04 (0.26)	0.0	**0.0096/0.020**
	C/G‐C/C	350	43.88 (0.17)	0.84 (0.24; 1.44)	
REC	G/G‐C/G	401	43.61 (0.15)	0.0	0.604/0.274
	C/C	100	43.69 (0.35)	0.08 (−0.62; 0.77)	
OVER	G/G‐C/C	251	43.29 (0.21)	0.0	0.050/0.211
	C/G	250	43.96 (0.19)	0.66 (0.11; 1.21)	
Log‐additive 0,1,2				0.39 (−0.01; 0.78)	**0.046/0.032**

*Note:* COD = codominant model (G/G vs. C/G vs. C/C; reference = G/G); DOM = dominant model (C/G–C/C vs. G/G; reference = G/G); REC = recessive model (C/C vs. G/G–C/G; reference = G/G–C/G); OVER = overdominant model (C/G vs. G/G–C/C; reference = G/G–C/C); log‐additive (0,1,2) = per‐allele effect coded by C‐allele dosage. Bold values indicate statistically significant results at *p* < 0.05.

Abbreviations: 95% CI = 95% confidence interval, Dif = mean difference, Me = estimated marginal mean, *n* = sample size, SE = standard error.

^a^ Values before the slash represent *p* values based on rank‐transformed data; values after the slash represent *p* values adjusted for age, BMI, and sex.

For *TNF‐α* rs1800629, grouped analyses suggested associations with both fitness status and LDL cholesterol. In the HF/LF comparison, carriers of the A allele showed lower odds of belonging to the HF group under the dominant model (OR = 0.55, 95% CI: 0.37–0.82; adjusted *p* = 0.048), while the strongest grouped signal was observed in the overdominant model, where heterozygotes (A/G) were less likely to belong to the HF group than homozygotes combined (OR = 0.51, 95% CI: 0.33–0.77; adjusted *p* = 0.030; Table 8). A similar pattern was observed for LDL cholesterol in the grouped sample, with the clearest association again seen under the overdominant model, where A/G individuals had higher LDL levels than homozygotes combined (difference = 7.21 mg·dL^−1^, 95% CI: 0.21–14.20; adjusted *p* = 0.011; Table 9). However, in whole‐cohort analyses, the association between *TNF‐α* rs1800629 and LDL cholesterol was attenuated and no model remained statistically significant after adjustment for age, BMI, and sex (Table 10).

**TABLE 8:** Association of *TNF‐α* rs1800629 with fitness status (LF vs. HF).

Model		LF N (%)	HF N (%)	OR (95% CI)	*p* ^a^
COD	G/G	126 (66.3)	243 (78.1)	1.00	**0.006**/0.090
	A/G	60 (31.6)	59 (19.0)	0.51 (0.34; 0.78)	
	A/A	4 (2.1)	9 (2.9)	1.17 (0.35; 3.86)	
DOM	G/G	126 (66.3)	243 (78.1)	1.00	**0.004/0.048**
	A/G‐A/A	64 (33.7)	68 (21.9)	0.55 (0.37; 0.82)	
REC	G/G‐A/G	186 (97.9)	302 (97.1)	1.00	0.585/0.660
	A/A	4 (2.1)	9 (2.9)	1.39 (0.42; 4.56)	
OVER	G/G‐A/A	130 (68.4)	252 (81.0)	1.00	**0.001/0.030**
	A/G	60 (31.6)	59 (19.0)	0.51 (0.33; 0.77)	
Log‐additive 0,1,2		190 (37.9)	311 (62.1)	0.66 (0.46; 0.93)	**0.019**/0.106

*Note:* COD (codominant: G/G vs. A/G vs. A/A; reference = G/G), DOM (dominant: A/G–A/A vs. G/G; reference = G/G), REC (recessive: A/A vs. G/G–A/G; reference = G/G–A/G), OVER (overdominant: A/G vs. G/G–A/A; reference = G/G–A/A), log‐additive (0,1,2) = per‐allele effect coded by A‐allele dosage. Bold values indicate statistically significant results at *p* < 0.05.

^a^ *p* values based on rank‐transformed values.

**TABLE 9:** Association of *TNF‐α* rs1800629 with LDL cholesterol (mg·dL^−1^) in the VO_2_max‐defined high fitness and low fitness groups.

Model		*n*	Me (SE)	Dif (95% CI)	*p* ^a^
COD	G/G	368	115.5 (1.8)	0.0	**0.037/0.029**
	A/G	119	122.4 (3.2)	6.92 (−0.10; 13.95)	
	A/A	13	107.2 (7.3)	−8.28 (−27.09; 10.53)	
DOM	G/G	368	115.5 (1.8)	0.0	**0.035/0.039**
	A/G‐A/A	132	120.9 (3.0)	5.43 (−1.34; 12.20)	
REC	G/G‐A/G	487	117.2 (1.6)	0.0	0.411/0.300
	A/A	13	107.2 (7.3)	−9.97 (−28.76; 8.81)	
OVER	G/G‐A/A	381	115.2 (1.7)	0.0	**0.012/0.011**
	A/G	119	122.4 (3.2)	7.21 (0.21; 14.20)	
Log‐additive 0,1,2				3.11 (−2.78; 8.99)	0.115/0.145

*Note:* Values before the slash represent *p* values based on rank‐transformed data; values after the slash represent *p* values adjusted for age, BMI, and sex. COD = codominant model (G/G vs. A/G vs. A/A; reference = G/G); DOM = dominant model (A/G–A/A vs. G/G; reference = G/G); REC = recessive model (A/A vs. G/G–A/G; reference = G/G–A/G); OVER = overdominant model (A/G vs. G/G–A/A; reference = G/G–A/A); log‐additive (0,1,2) = per‐allele effect coded by A‐allele dosage. Bold values indicate statistically significant results at *p* < 0.05.

Abbreviations: 95% CI = 95% confidence interval, Dif = mean difference, Me = estimated marginal mean, *n* = sample size, SE = standard error.

^a^ *p* values based on rank‐transformed values, after slash—*p* values adjusted for age, BMI and sex.

**TABLE 10:** Association of *TNF‐α* rs1800629 with LDL cholesterol (mg·dL^−1^) in the whole cohort.

Model	*n*	Me	Se	Dif	Lower	Upper	*p* ^a^
Codominant							
G/G	691	112.18	1.25	0.00			0.110/0.187
A/G	246	116.15	2.31	3.97	−0.92	8.87	
A/A	23	103.43	5.29	−8.74	−22.72	5.23	
Dominant							
G/G	691	112.18	1.25	0.00			0.233/0.146
A/G‐A/A	269	115.06	2.17	2.89	−1.86	7.63	
Recessive							
G/G‐A/G	937	113.22	1.11	0.00			0.169/0.478
A/A	23	103.43	5.29	−9.78	−23.71	4.14	
Overdominant							
G/G‐A/A	714	111.89	1.22	0.00			0.087/0.081
A/G	246	116.15	2.31	4.26	−0.62	9.13	
Log‐additive							
0,1,2				1.36	−2.82	5.55	0.524/0.287

*Note:* COD (codominant: G/G vs. A/G vs. A/A; reference = G/G), DOM (dominant: A/G–A/A vs. G/G; reference = G/G), REC (recessive: A/A vs. G/G–A/G; reference = G/G–A/G), OVER (overdominant: A/G vs. G/G–A/A; reference = G/G–A/A), log‐additive (0,1,2) = per‐allele effect coded by A‐allele dosage.

Abbreviation: *n* = sample size.

^a^ *p* values based on rank‐transformed values, after slash—*p* values adjusted for age, BMI and sex.

### 3.2 Multi‐Locus Analysis—IL6 Haplotypes and Targeted Gene–Gene Interactions

We estimated haplotypes from the three *IL6* SNPs and tested haplotype–phenotype association. In the *IL6* locus (rs1800795–rs1800796–rs1800797), six haplotypes were observed. The most frequent haplotype was G–G–G (frequency 0.486), followed by C–G–G (0.336), C–G–A (0.114), and G–C–G (0.063). Two haplotypes were rare (G–G–A, 0.0011; C–C–G, ~0) and were excluded from haplotype‐based association models. The G–G–G haplotype was used as the reference in regression analyses.

Among four targeted phenotypes showing single‐locus evidence of association with at least one of the *IL6* variants (i.e., iron, HCT, HGB, RBC), the *IL6* haplotype block showed a significant global association with iron (FDR‐adjusted *p* (Q) = 0.032), whereas the association with hematocrit was not significant (Q = 0.084). For iron, follow‐up haplotype‐specific regression (reference G–G–G; rare haplotypes < 1% excluded) C‐G‐G haplotype was associated with higher iron (β = 13.9, SE = 4.27, *p* = 0.0012; FDR‐adjusted within‐phenotype *p* = 0.002). The remaining common haplotypes (C‐G‐A and G‐C‐G) were not significantly associated with iron after within‐phenotype FDR correction (*p* = 0.633). In the exploratory screening, we applied the *IL6* haplotype omnibus test (df = 4) across 43 continuous phenotypes, excluding haplotypes with frequency < 1%. After Benjamini–Hochberg FDR correction across all exploratory phenotypes, no associations remained significant (minimum FDR‐adjusted *p* = 0.976 for creatinine). Several traits showed nominal evidence (raw *p* < 0.05)—notably serum creatinine (*p* = 0.033) and platelets (PLT) (*p* = 0.045)—but none survived multiple‐testing correction (thus no haplotype‐specific models were fitted for these phenotypes). Complete results of the exploratory haplotype‐based analysis are presented in Table S7.

Targeted gene–gene interaction analyses were performed for selected phenotypes showing single‐locus signals (iron, HCT, LDL, and mean VO_2_max). For each phenotype, we added a block of *IL6* × *IL15* interaction terms, and separately a block of *IL6* × *TNF‐α* interaction terms, using the most frequent non‐reference *IL6* haplotypes (> 1% for continuous and > 10% for VO_2_max), and compared models with and without these terms using block likelihood‐ratio tests. Neither interaction block (*IL6 × IL15* and *IL6 × TNF‐α*) reached statistical significance (Table S8).

**FIGURE 1:**
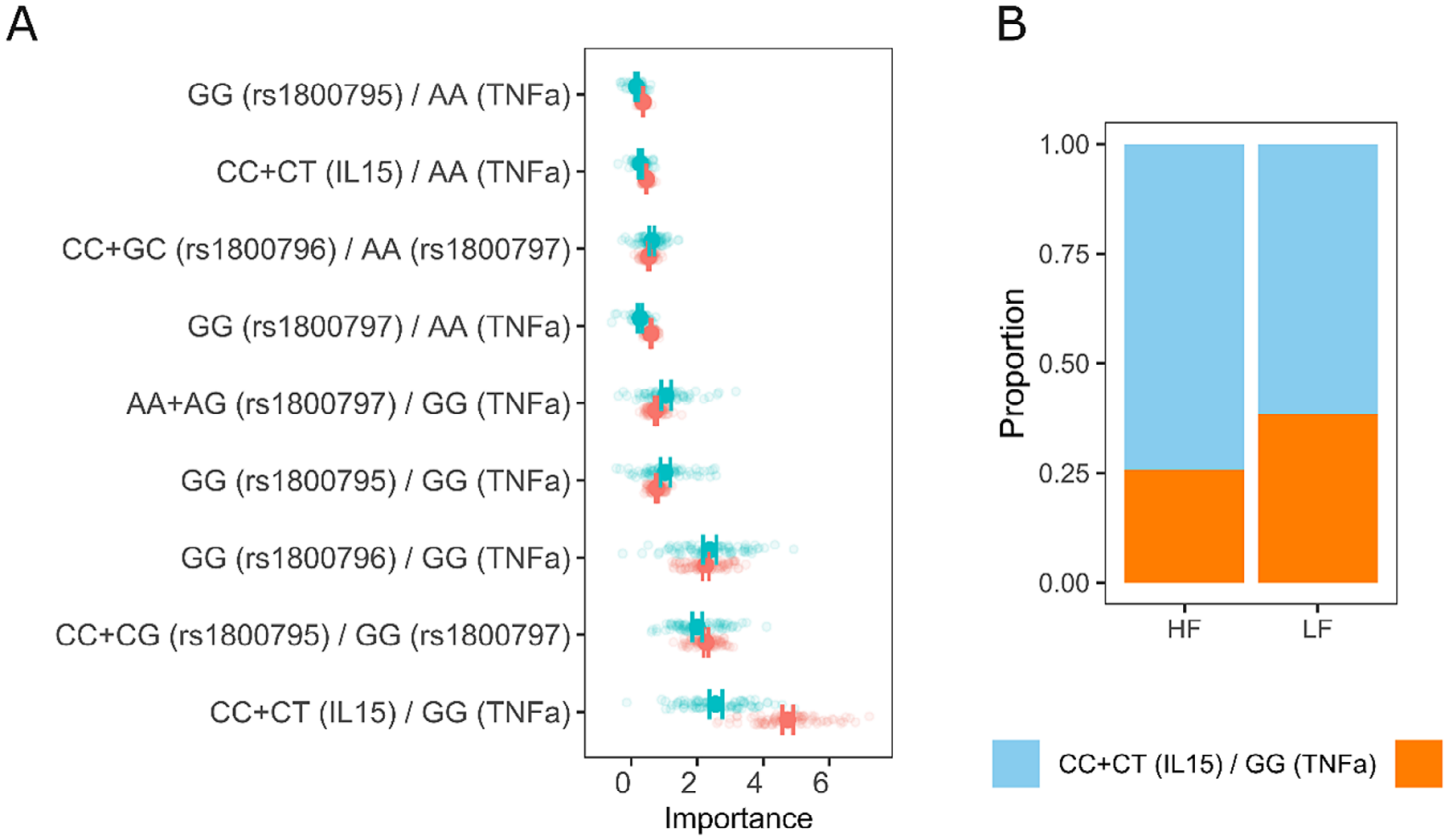
Data‐driven multi‐locus analysis (*IL6*, *IL15*, *TNF‐a*) association analysis with VO_2_max status. (A) Importance scores for genotype combinations across 100 runs on real data (red) and 100 runs on phenotype‐resampled (VO_2_max‐shuffled, turquoise) data. Median and bootstrap 95% CIs are shown. (B) Frequency of the top combination (*IL15* CC + CT with *TNF‐α* GG) in high‐fitness (HF) versus low‐fitness (LF) groups for the real dataset (χ^2^ *p* = 0.004).

To further explore potential non‐additive and higher‐order interactions between loci beyond the predefined haplotype and pairwise interaction framework, we extended the analysis using a model‐free approach (logic regression) to jointly evaluate SNPs from the *IL6*, *IL15*, and *TNF* loci in relation to VO_2_max (Figure 1).

Multi‐locus logic regression analysis identified the genotype combination *IL15* CC + CT and *TNF*‐α GG as the most influential, with a median importance of 4.74 (95% CI: 4.57–4.91) in real data, compared to 2.55 (95% CI: 2.36–2.75) in resampled data (Figure 1A). This combination was significantly more frequent in the HF group than in the LF group (Chi‐squared *p* = 0.004, Figure 1B).

In summary, the most consistent single‐locus signal was the association of *IL6* rs1800795 with hematological traits, including serum iron, HCT, HGB, and RBC count, particularly in covariate‐adjusted whole‐cohort analyses. TNF‐α rs1800629 was associated with aerobic fitness classification in grouped analyses, whereas an IL15 × TNF‐α combination emerged in the exploratory logic‐regression analysis as a multi‐locus pattern associated with high‐fitness status.

## 4 Discussion

This study examined whether five SNPs in *IL6* (rs1800795, rs1800796, rs1800797), *IL15* (rs1589241), and *TNF‐α* (rs1800629) genes are associated with body composition, selected hematological and biochemical traits, and aerobic and anaerobic performance‐related phenotypes. We combined VO_2_max‐stratified HF/LF analyses in 501 participants with selected whole‐cohort analyses of hematological and biochemical traits in up to 962 participants. A major strength of this study is the rigorous, multi‐stage phenotyping strategy applied to a large initially recruited cohort, resulting in a well‐characterized cohort (up to 962 adults) with outcome‐specific availability of physiological, biochemical, and genomic data. Another important strength lies in the innovative classification strategy; instead of comparing athletes with non‐athletes, participants were grouped according to objectively measured aerobic capacity (VO_2_max) based on standardized norms. In addition, selected whole‐cohort analyses enabled us to examine trait‐level associations beyond dichotomized fitness status. When combined with the analysis of five single‐locus polymorphisms, haplotypes, and gene–gene interactions, these features constitute a unique contribution of this study to the field.

When comparing basic physiological and biochemical variabilities between the participants with very good or outstanding VO_2_max and participants with satisfactory, poor, or very poor VO_2_max, clear differences were observed. Participants who had higher VO_2_max values exhibited healthier lipid profiles and lower white blood cell counts, consistent with a more favorable physiological profile. They also presented with lower body mass and waist circumference, reflecting more favorable metabolic health. Interestingly, although their red blood cell and hemoglobin levels were significantly lower than those observed in the LF group, the HF group demonstrated higher mean corpuscular volume (MCV), and mean corpuscular hemoglobin (MCH), indicating fewer but larger erythrocytes, which may reflect differences in hematological phenotype associated with fitness level. Our findings regarding erythrocyte size and blood volume may be related to fitness‐associated physiological differences, with a possible contributory genetic component. Enhanced red blood cell (RBC) deformability and reduced RBC density have been shown to be advantageous for endurance performance and are, in part, a consequence of increased RBC turnover (Bizjak et al. 2020). Based on these observations, it can be hypothesized that the *IL6* rs1800795 polymorphism may influence serum iron availability. This remains an association only. Directional causality cannot be inferred from cross‐sectional data.

Numerous studies have confirmed significant training‐induced changes in body mass and composition, as well as in physiological and biochemical parameters. These typically include reductions in body weight, fat mass, and skinfold thickness; increases in VO_2_max and cardiorespiratory efficiency; favorable alterations in hematological indices (HCT, MCV, MCHC, WBC, neutrophils, monocytes, MPV); and improvements in glucose homeostasis and lipid profile (Bachero‐Mena et al. 2017; Kostrzewa‐Nowak et al. 2015; Leońska‐Duniec et al. 2024). In addition, regular exercise is associated with reduced resting heart rate and blood pressure, improved endothelial function, increased muscle capillarization and mitochondrial density, as well as favorable modulation of inflammatory and oxidative stress markers (e.g., CRP, IL6, TNF‐α) (Carlson et al. 2014; Granata et al. 2018; Mølmen et al. 2025). These findings raise the question of whether genetic variation, particularly in cytokine regulation, may help explain these patterns of exercise‐related physiological and biochemical differences. In this context, the High Fitness group displayed lower WBC and a markedly more favorable HDL/LDL/TG profile alongside superior aerobic and anaerobic metrics, which provides a phenotypic backdrop for testing cytokine‐pathway genetics. Because the study was cross‐sectional and detailed training‐history data were unavailable, these between‐group differences should not be interpreted as demonstrated training adaptations or as evidence of genotype‐driven physiological effects.

To examine the influence of these genetic variables, firstly three functional polymorphisms (rs1800795, rs1800796, rs1800797) located in the promoter region of the *IL6* gene were investigated. All these variants have been suggested to influence IL6 expression (Yadav et al. 2024) and consequently modulate post‐exercise inflammation and recovery (Yadav et al. 2024; Nash et al. 2023). For example, the C allele of rs1800795 (−174G/C) has been associated with significantly reduced IL6 serum concentrations in response to long‐term exercise training programs (Oberbach et al. 2008). Although grouped VO_2_max analysis did not reveal significant associations between individual or combined *IL6* polymorphisms and fitness category itself, genotype‐specific differences in blood parameters were observed. Individuals with the CC genotype of rs1800795 exhibited slightly higher hematocrit and iron levels. In the expanded whole‐cohort analyses adjusted for age, BMI, and sex, rs1800795 showed the most consistent associations with serum iron, hematocrit, hemoglobin, and RBC count, indicating that *IL6*‐related variation in this cohort was linked more strongly to hematological traits than to dichotomized VO_2_max status itself. In contrast, the other two *IL6* promoter polymorphisms showed no significant associations with the blood‐based markers assessed. However, the *IL6* haplotype block showed a significant global association with iron, whereas the association with hematocrit was not significant. Specifically, the C‐G‐G (rs1800795–rs1800796–rs1800797) haplotype was associated with higher iron, while other common haplotypes (C‐G‐A and G‐C‐G) were not significant. These genetic findings complement evidence from non‐genetic markers, such as cfDNA responses to maximal exercise (Humińska‐Lisowska et al. 2021) and adipokine dynamics modulated by fat mass and exercise intensity (Humińska‐Lisowska et al. 2022), both highlighting the role of inflammatory and metabolic axes in shaping recovery. Consistent with our findings, other authors have confirmed the association of the rs1800795 polymorphism with various biochemical blood parameters. Specifically, the CC genotype has been linked to lower circulating IL6 levels and reduced systemic inflammation (Abuelnadar et al. 2024), as well as to differences in glucose metabolism and lipid profile in several cohorts (Leońska‐Duniec et al. 2024; Halverstadt et al. 2005). In addition, recent evidence indicates that IL6 signaling contributes to iron metabolism through the IL6/JAK1/STAT3 pathway, which upregulates divalent metal transporter 1 (*DMT1*) expression via increased hypoxia‐inducible factor‐1 α (HIF‐1α) activity (Xie et al. 2024). Although circulating IL‐6 concentrations were not measured in this study, these literature‐based mechanisms therefore only provide biological context. A link between genotype, IL‐6 expression, and iron homeostasis cannot be inferred from the present cross‐sectional data. Taken together, our findings suggest that *IL6* variation may contribute modestly to the hematological and metabolic background of exercise phenotype, even in the absence of a direct association with VO_2_max category.

Secondly, the rs1800629 polymorphism located in the proximal promoter region of *TNF‐α* was examined. *TNF‐α* has a dual role in skeletal muscle: brief signaling helps initiate myogenesis and tissue repair, whereas sustained or excessive TNF‐α drives NF‐κB‐ubiquitin‐proteasome catabolism, promotes chronic inflammation, and delays recovery—ultimately impairing performance (Chen et al. 2007; Tidball 2017). In the present grouped analysis, carriers of the A allele had lower odds of belonging to the HF group under the dominant model, whereas the strongest association was observed for AG heterozygotes under the overdominant model. This indicates a model‐dependent association with grouped aerobic fitness status and should not be interpreted as a direct effect of the A allele on performance. The rs1800629 A allele has been associated in other settings with increased TNF‐α transcriptional activity, particularly under conditions of stress or immune activation (AbdElneam et al. 2024).

Elevated TNF‐α has been reported to impair endurance‐related adaptations by inducing skeletal‐muscle insulin resistance, suppressing protein synthesis, and perturbing myogenesis, thereby delaying recovery (Lang et al. 2002; Plomgaard et al. 2005; Shirakawa et al. 2021). Previous studies have also reported associations of rs1800629 with smaller CRP reductions in response to physical activity in A‐allele carriers (Kilpeläinen et al. 2010) and with physical‐function performance or training‐related biochemical changes in other populations (Yao et al. 2020; Leońska‐Duniec et al. 2019). These observations provide biological context for the grouped association observed here. However, circulating TNF‐α concentrations and transcriptional activity were not measured in our cohort, and the present data do not establish that the observed association is mediated by TNF‐α expression.

In our grouped analyses, *TNF‐α* rs1800629 also showed an association with LDL cholesterol, with the clearest signal in the overdominant model; however, this association was attenuated in whole‐cohort analyses and no model remained statistically significant after adjustment for age, BMI, and sex. Beyond aerobic capacity, we observed an unadjusted, suggestive trend for longer Wingate time to peak power in AA versus AG/GG (*p* = 0.052) (Table S7). Although the direction of this trend is consistent with prior experimental findings that elevated TNF‐α can impair excitation–contraction coupling and recovery (Hardin et al. 2008; Pereira et al. 2013; Reid and Li 2001), this interpretation remains speculative in the absence of direct cytokine measures. Taken together, the *TNF‐α*‐related findings were model‐dependent and not uniformly consistent across analytical frameworks. They should therefore be regarded as preliminary associations rather than evidence that the A allele is disadvantageous for aerobic or anaerobic performance within this modeling analyses.

Together, these findings suggest that cytokine‐related polymorphisms influence distinct components of the exercise response—*IL6* variants linked primarily to iron handling and hematological adaptation, and *TNF*‐α variants associated with grouped fitness status, with less consistent evidence for lipid‐related effects across all analyses. The broader research program from which this analysis derives also examines circulating biomarkers and microbiome features. These complementary data were not analyzed here and do not provide direct functional validation of the present genetic associations (Humińska‐Lisowska et al. 2025). These observations support a broader multi‐factorial model of exercise phenotype, but do not by themselves justify a strong inflammatory‐genetic‐microbial interpretation.

The next polymorphism examined was rs1589241, located in a regulatory intronic region of the *IL15* gene, which encodes an important exercise‐induced cytokine involved in immune regulation, fat metabolism, mitochondrial biogenesis, and skeletal muscle growth (O'Leary et al. 2017; Pistilli et al. 2008; Pistilli and Quinn 2013). Our single‐locus analysis revealed no significant associations between the *IL15* genotypes and VO_2_max or biochemical variabilities. However, notable findings emerged when gene–gene interactions were modeled using exploratory logic‐regression analysis. Specifically, individuals carrying the *IL15* CC or CT genotypes together with the *TNF‐α* GG genotype were significantly more likely to be classified as participants with higher VO_2_max. This exploratory multi‐locus pattern was associated with HF status, but it does not establish a biological interaction, cytokine‐expression profile, recovery phenotype, or training‐induced adaptation. Biological plausibility for *IL15* in endurance adaptation is strong, as endurance training has been shown to upregulate muscular *IL15* in humans, promoting myogenesis and mitigating inflammation‐induced atrophy (O'Leary et al. 2017; Pistilli and Quinn 2013; Rinnov et al. 2014).

Although the interaction between these genes and VO_2_max has not been studied so far, the relationship between these cytokines is well described. IL15 and TNF‐α exert opposing influences on skeletal muscle: TNF‐α promotes catabolic signaling and impairs contractile function, whereas IL15 is a myokine with pro‐myogenic and anti‐atrophic actions. Notably, human myotubes secrete IL15 in response to TNF‐α, consistent with a compensatory, anti‐inflammatory feedback loop (Reid and Li 2001; O'Leary et al. 2017). IL15 attenuates TNF‐α–driven proteolysis and apoptosis in muscle and can inhibit downstream catabolic TNF‐α pathways (e.g., NF‐κB/UPS), thereby preserving muscle mass and function (Pistilli and Quinn 2013; Liu et al. 2015).

In athletes, endurance training increases skeletal‐muscle IL15 protein content, positioning IL15 as a training‐responsive mediator of adaptation (Rinnov et al. 2014). Conversely, dampening TNF‐α signaling facilitates recovery after injury, underscoring the performance cost of a pro‐inflammatory environment associated with elevated TNF‐α (Pistilli and Quinn 2013; Rinnov et al. 2014; Stratos et al. 2022). The cited literature provides biological context for considering *IL15* and *TNF‐α* jointly; however, the present multi‐locus result is exploratory and hypothesis‐generating. Replication in independent cohorts with direct cytokine measurements is required before any mechanistic interpretation is made.

Collectively, these findings highlight the potential value of integrating genetic markers with physiological and biochemical profiling when investigating individual variability in exercise phenotype. While current effect sizes are modest, such integrative approaches may help refine mechanistic understanding of exercise‐related phenotypes. However, the present results should be regarded as hypothesis‐generating rather than immediately applicable to training personalization, recovery monitoring, or targeted interventions in athletic or clinical settings.

## 5 Limitations

There are several limitations of this study that should be acknowledged. The cross‐sectional, single‐time‐point design precludes drawing causal inferences about training responses or long‐term adaptations. Circulating concentrations *of IL6*, *IL15*, and *TNF‐α*, transcriptomic (RNA expression), or proteomic (e.g., receptor abundance/signaling markers or circulating proteins) were not measured. Therefore, any interpretation linking genotype to cytokine expression, signaling, or exercise‐related biological mechanisms remains inferential. Although selected analyses were adjusted for age, sex, and BMI, detailed information on habitual physical activity, long‐term training exposure, prior competitive athletic status, previous fitness level, dietary intake, and smoking status was not comprehensively available and could not be included as covariates. These factors may have contributed to residual confounding. In addition, the HF/LF comparison was based on dichotomized VO_2_max categories, and participants classified as medium or good were excluded from this analysis. This design maximized phenotypic contrast but reduced sample size and does not represent the full continuum of aerobic fitness. The study represented a single‐country cohort of adults of Polish European ancestry, which limits generalizability to other ancestries, regions, and sport populations. Replication in more diverse, multi‐center cohorts is needed. A candidate‐gene approach was followed focused on biologically plausible *loci*, but it does not capture genome‐wide genetic variation. Thus, the contribution of other variants remains unexplored. Finally, rare genotype groups, particularly *TNF‐α* rs1800629 AA, limited power in some inheritance models, and deviations from Hardy–Weinberg equilibrium for *IL15* rs1589241 and *IL6* rs1800797 in the pooled sample, and *TNF‐α* rs1800629 in the HF group, require cautious interpretation of findings involving these variants. Prospective, longitudinal, and independently replicated studies are needed.

## 6 Conclusions

Taken together, the results of this study provide a multilevel picture of how cytokine‐related genes may potentially contribute to variability in fitness‐related physiological and biochemical traits. In the VO_2_max‐stratified sample of 501 adults, complemented by selected outcome‐specific whole‐cohort analyses of up to 962 participants, *IL6* rs1800795 was associated primarily with iron‐related and hematological traits, including hematocrit, hemoglobin, and RBC count. *TNF‐α* rs1800629 showed a model‐dependent association with lower aerobic fitness classification and an unadjusted trend toward slower anaerobic power development, reflected by longer time to reach peak power in AA homozygotes. Importantly, an exploratory multi‐locus pattern was also observed: the *IL15* CC/CT × *TNF‐α* GG combination was associated with greater odds of higher aerobic fitness classification. These results highlight the potential value of considering gene–gene combinations alongside single‐variant models and integrating genetic markers with physiological profiling. Because several signals were modest or exploratory, and the study is cross‐sectional in a European cohort, replication in independent cohorts with rigorous covariate adjustment, multiplicity control, detailed lifestyle and training‐history data, and direct cytokine measurements is warranted to define effect sizes and generalizability.

## Author Contributions

K.H.‐L. participated in the conceptualization and supervision of the study, contributed to data curation, formal analysis, funding acquisition, investigation, methodology, project administration, resources, software, validation, visualization, writing – original draft and review and editing; P.A. contributed to data curation, formal analysis, investigation, methodology and resources; A.L.‐D. contributed to data curation and writing – original draft and review and editing: B.B. contributed to investigation, methodology, validation, visualization and writing – review and editing; M.M.‐S. contributed to investigation, methodology, project administration and validation; A.V.S. contributed to software and writing – review and editing; P.P. contributed to visualization, validation and writing review and editing. All authors have read and approved the final version of the manuscript and agree with the order of presentation of the authors.

## Funding

This work was co‐financed by Gdańsk University of Physical Education and Sport through an internal research project led by K.H.‐L. as Principal Investigator, and from the state budget of the Republic of Poland under the programme of the Minister of Education and Science entitled “Science for Society II” (project no. NdS‐II/SP/0503/2024/01). A.V.S. was supported by the South African Medical Research Council through its Division of Research Capacity Development under the Mid‐Career Scientist Programme.

## Ethics Statement

The study complied with the Declaration of Helsinki and was approved by the Bioethics Committee at the District Medical Chamber in Gdańsk (KB‐16/20).

## Conflicts of Interest

The authors declare no conflicts of interest.

## Supporting information


**Table S1:** Aerobic capacity standards based on VO_2max_ levels adapted from Shvarz and Reibold, 1990.
**Table S2:** IL6 1,800,795 (*N* = 501).
**Table S3:** IL6 1,800,796 (*N* = 501).
**Table S4:** IL6 1,800,797 (*N* = 501).
**Table S5:** IL15 rs1589241 (*N* = 501).
**Table S6:** TNFa rs1800629 (*N* = 501).
**Table S7:** IL6 haplotype global (omnibus) associations for exploratory phenotypes.
**Table S8:** Interaction block (IL6 × IL15 and IL6 × TNFα) *p* values from comparing of nested models with and without the interaction term.

## Data Availability

All data produced in the present study are available upon reasonable request to the authors.
